# Suction‐feeding performance in invasive alien giant salamanders within the functional framework of *Andrias*

**DOI:** 10.1111/joa.70262

**Published:** 2026-10-24

**Authors:** M. López‐Pachón, S. Hayashi, J. Fortuny, J. Marcé‐Nogué

**Affiliations:** ^1^ Department d'Enginyeria Mecànica Universitat Rovira i Virgili Tarragona Spain; ^2^ Department of Dinosaur Paleontology, Faculty of Biosphere‐Geosphere Okayama University of Science Okayama Okayama Prefecture Japan; ^3^ The University of Osaka Suita Osaka Prefecture Japan; ^4^ Institut Català de Paleontologia Miquel Crusafont (ICP‐CERCA), Universitat Autònoma de Barcelona, Edifici ICTA‐ICP, c/ de les Columnes s/n Barcelona Catalonia Spain

**Keywords:** *Andrias* cf. *davidianus*, *Andrias japonicus*, conservation biology, cryptobranchid, functional morphology, high‐speed videography, hybridisation, prey capture, suction feeding

## Abstract

Hybridisation between *Andrias japonicus* and *A.* cf. *davidianus* lineages is a major conservation concern in Japan, but whether hybrid individuals retain the suction‐feeding pattern described for non‐hybrid *Andrias* remains unclear. We analysed suction feeding in six hybrid giant salamanders using 20 captive feeding events recorded with lateral and frontal high‐speed videography. Skull, lower jaw, hyobranchial and prey movements were digitised to quantify trajectories, velocities and accelerations during the active expansion phase of prey capture. The hybrids displayed the characteristic suction‐feeding sequence described for cryptobranchids, including mandibular opening, hyobranchial expansion, prey acceleration and capture once suction was initiated. Event‐level variation occurred in relation to prey position, attack configuration and individual morphometrics, highlighting the flexibility of suction feeding performance among hybrid specimens. Overall, hybrid specimens retained suction‐feeding kinematics within the functional pattern previously described for *Andrias*, suggesting that hybridisation does not disrupt the core biomechanical organisation of prey capture within the functional range examined here. When compared with published data for non‐hybrid *A. davidianus* and *A. japonicus*, these results indicate a broadly conserved suction feeding mechanism rather than a distinct hybrid‐specific feeding mode. Therefore, any potential ecological advantage of hybrids is unlikely to result from a fundamental change in the suction feeding sequence alone.

## Graphical abstract

Using high‐speed video, we quantify suction‐feeding variability in hybrids of *Andrias* japonicus × A. cf. davidianus. Body condition and attack geometry modulate prey kinematics, revealing a flexible suction mechanism with ecological and conservation implications.

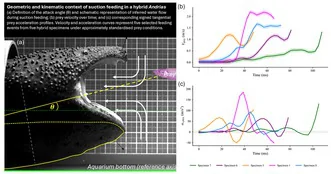

## 1 INTRODUCTION

Cryptobranchids, including the genus *Andrias*, represent one of the most ancient lineages of giant amphibians. The genus *Andrias* is known from the fossil record since the Oligocene (e.g. Böhme, 2008; Westphal, 1958) and has retained a remarkably conserved morphology. Their fully aquatic lifestyle, together with the structure of the skull and the hyobranchial apparatus, enables a specialised suction feeding mechanism (Heiss et al., 2013; Matsumoto et al., 2024). During this process, the rapid expansion of the buccopharyngeal cavity generates negative pressures that accelerate water and prey into the mouth (Schwarz et al., 2023; Spence et al., 2023).

In Japan, *Andrias japonicus* currently faces a significant threat as a result of hybridisation with *A. davidianus* (Yoshikawa et al., 2011). This introduced lineage corresponds to the Chinese giant salamander, which was translocated into several Japanese regions without regulation during recent decades (Matsui, 2006). This situation is widely recognised as a serious conservation concern because it alters the genetic integrity of native populations. Recent geometric morphometric analyses show that hybrid specimens display cranial shapes that are intermediate between those of the two parental species (Hara et al., 2025). Cranial morphology may also have relevance beyond feeding, as biting plays an important role in male–male combat in *A. japonicus* (Takahashi et al., 2024). These findings raise a functional question: how hybrid cranial morphology is expressed in suction‐feeding performance relative to the functional range known for non‐hybrid *Andrias*.

Recent anatomical and biomechanical studies have shown that the evolution of cryptobranchid feeding is closely linked to cranial flattening, hyobranchial development, asymmetric jaw mechanics and hydrodynamic suction performance, as documented through CT‐based cranial reconstruction, high‐speed video, finite element analysis, computational fluid dynamics and digitisation‐based modelling (Fortuny et al., 2015; Heiss et al., 2013; Kolchanov & Skutschas, 2026; López‐Pachón & Marcé‐Nogué, 2025). In this functional context, suction feeding is also associated with asymmetric jaw movements and coordinated skull, mandibular and hyobranchial motion, providing a comparative baseline for interpreting feeding performance in *Andrias* (Cundall et al., 1987; Matsumoto et al., 2024).

Available studies suggest that both parental species share the same general suction‐feeding organisation, although different functional features have been emphasised. In *A. davidianus*, prey capture is strongly jaw‐powered: rapid separation of the broad jaws accelerates water and prey before the main contribution of hyobranchial depression, and feeding performance is influenced by prey position and jaw loading (Fortuny et al., 2015; Heiss et al., 2013). In *A. japonicus*, feeding likewise involves coordinated mandibular and hyobranchial motion, but pronounced left–right asymmetry related to prey position has been documented, enabled by mobility of the quadrate–articular joint and mandibular symphysis (Cundall et al., 1987; Matsumoto et al., 2024). Thus, *Andrias* appears to share a conserved suction‐feeding organisation while allowing variation in cranial and hyobranchial function. This is particularly relevant to hybrids, which show an overall intermediate cranial morphology but also some distinctive features, including a wider skeletal mouth than either parental species (Hara et al., 2025). Such morphology may influence prey capture and the space available for the hyobranchial apparatus, potentially modifying kinematic timing or magnitude without requiring a fundamentally different feeding mechanism.

Within this form–function framework, the hybrid specimens analysed here provide an opportunity to examine whether changes in cranial form are associated with measurable differences in suction‐feeding function. The present study therefore documents for the first time the suction‐feeding mechanism in hybrid *Andrias* specimens to address one specific question: whether hybrids retain the basic suction‐feeding organisation described for non‐hybrid *Andrias* while showing variation in the timing and magnitude of prey‐capture kinematics. Direct trials with parental species were beyond the scope of this study; however, available studies of their suction mechanics provide a comparative baseline.

To address this question, we combined high‐speed kinematic analysis, morphological characterisation and hydrodynamic interpretation. Specifically, we focus on the coordinated motion of cranial, mandibular and hyobranchial elements, together with prey motion, to define the active expansion phase of suction feeding and its anatomical correlates. Secondarily, we assess whether morphology, body condition and attack configuration may contribute to within‐group variation in suction‐feeding performance.

## 2 MATERIALS AND METHODS

### 2.1 Specimens

A total of 11 hybrid specimens of the genus *Andrias* were used in this study. According to official documentation from the Kagamino Town Board of Education, the specimens originated from the Yoshii River in Tomata District (Okayama Prefecture, Japan), where they were collected and subsequently transferred to a dedicated quarantine facility.

Available records identified the animals as hybrids between *Andrias japonicus* and *A*. cf. *davidianus*. Here, ‘cf.’ indicates taxonomic affinity with *A. davidianus*, while acknowledging uncertainty in the precise identity of the introduced Chinese lineage. However, raw genetic data and detailed pedigree information were not available for the present study. Therefore, although some records classify these individuals as F2 hybrids, we refer to them here conservatively as hybrid *Andrias* specimens and do not assign them to a specific hybrid generation. This uncertainty is acknowledged as a limitation because hybrid generation, backcross ancestry or independent hybridisation events may influence cranial morphology and functional variation.

These animals were housed in individual aquaria under controlled environmental conditions. Water temperature was maintained at 15°C using a GEX Cool Way BK‐C220 cooling system, and water quality was ensured through an EHEIM 2213 filtration system. No specific adjustments to humidity were required due to the fully aquatic nature of the experiment. Outside of the experimental trials, *Andrias* specimens were maintained under the routine husbandry feeding regime applied by the animal‐care staff for cryptobranchids.

Before each filming session, food was withheld for 24 h to standardise feeding motivation and reduce the potential effects of recent feeding. According to the animal‐care staff, some individuals had not voluntarily fed during the preceding days under routine husbandry conditions; however, because individual feeding histories could not be verified consistently, these observations were not treated as experimentally controlled fasting intervals.

Body mass was recorded for each specimen at the time of the experiments. Sex could not be reliably determined for any specimen because external sexual dimorphism in *Andrias* is limited, being mainly restricted to cloacal swelling in males during the breeding season (Klein et al., 2024), and no invasive procedures were performed. Exact chronological age was not available for the animals; specimens were therefore characterised based on developmental stage and body size rather than age.

All procedures were conducted under permits issued by the Japanese authorities, including the Environmental & Land Management Permit No. 2412258 (Ministry of the Environment of Japan) and complementary authorisations from the Kagamino Town Board of Education for the use of hybrid salamanders originating from the Yoshii River (see Supporting Information Material). The study adhered to institutional animal‐welfare guidelines from Okayama University of Science, Nagoya University, the Kanagawa Prefectural Museum of Natural History (all of them, from Japan) and Universitat Rovira i Virgili (Spain).

### 2.2 Morphometrics and intraspecific variability

The specimens covered a size range within the available hybrid sample (Figure 1a), spanning variation in snout–vent length (SVL), snout–tail length (STL), head length (HL), head width (HW) and body mass (M), all measured prior to each experimental trial. From these measurements, two proportional indices were calculated: the head length to snout–vent length ratio (HL/SVL) and the relative head width (HW/HL). In addition, body condition was quantified using a body condition index (BCI), calculated as body mass divided by snout–vent length cubed, with SVL expressed in metres. This index was used as a size‐standardised proxy for body condition and was not interpreted as tissue density. Because individual feeding histories before the final 24 h food‐withholding period could not be consistently verified, fasting history was not included in the analyses.

**FIGURE 1:**
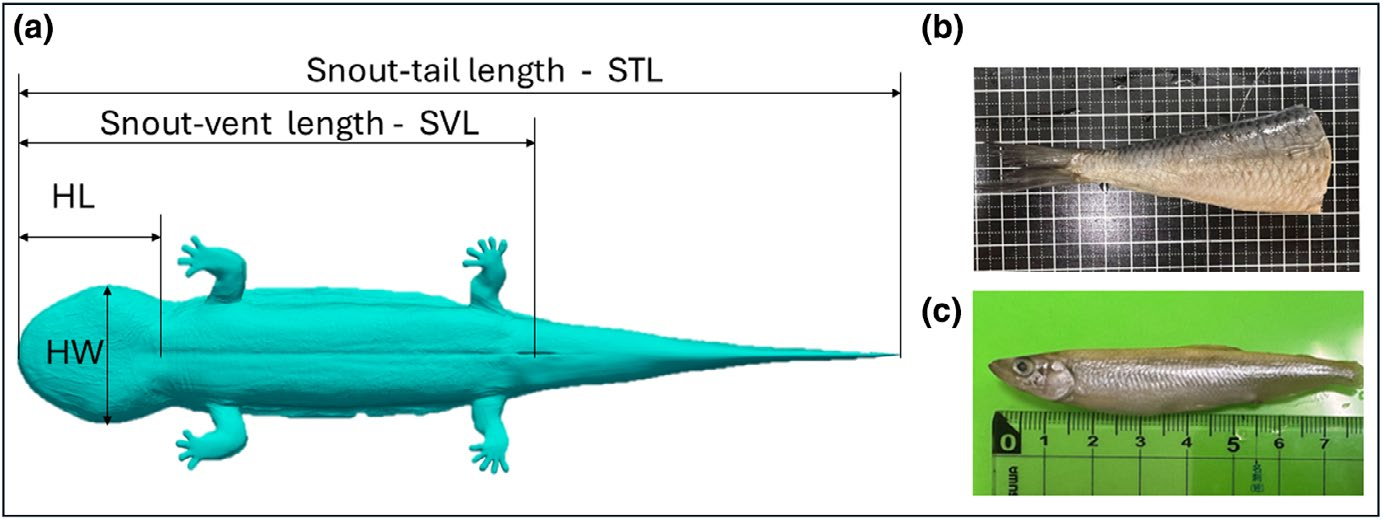
Morphometric measurements of hybrid *Andrias* specimens and prey items used in the feeding trials. (a) Definition of snout–vent length (SVL), snout–tail length (STL), head length (HL; measured from the snout tip to the posterior margin of the skull), and head width (HW; maximum transverse width of the head) in the salamander model. BCI was calculated as body mass divided by snout–vent length cubed, with SVL expressed in metres. (b) Horse mackerel (*Trachurus japonicus*) and (c) Japanese smelt (*Hypomesus nipponensis*). Salamander 3D model previously described by López‐Pachón and Marcé‐Nogué (2025).

Because sex could not be reliably determined for the hybrid specimens, they were classified as juveniles, subadults or adults based on overall size and body proportions, with reference to the cryptobranchid literature and previous anatomical studies (Kleinteich et al., 2014; Matsumoto et al., 2024). The complete set of morphometric measurements for the hybrid specimens is provided in Table S1.

### 2.3 Acclimation and behavioural considerations

*Andrias* exhibit very low locomotor activity and a sedentary lifestyle, traits typical of cryptobranchids with reduced metabolic rates (Klein et al., 2024). These features make them highly sensitive to disturbance, and moving specimens to a new tank often suppresses feeding (Zhang et al., 2006). For this reason, each salamander was given at least 12 h of acclimation in the tank used for filming, or alternatively the recording system was installed directly in its habitual aquarium.

Finally, given the predominantly nocturnal activity of *Andrias* (Matsui, 2014), feeding reactions were more reliable during afternoon sessions in our trials, whereas morning trials were rarely successful. Because *Andrias* salamanders show aggression and cannibalistic behaviour, especially between different‐sized specimens (Browne et al., 2014), acclimation was always performed with a single specimen per tank.

### 2.4 Experimental setup

Our methodological approach followed the foundational work of Heiss et al. (2013), who integrated high‐speed videography with detailed kinematic analyses to describe the mandibular dynamics and hydrodynamic patterns associated with suction feeding in *Andrias davidianus*. In the present study, we adapted this framework to the specific characteristics of the hybrid specimens and the available equipment, with the aim of accurately capturing rapid mandibular opening, hyoid displacement and prey transport into the buccopharyngeal cavity.

Feeding events were recorded in lateral and frontal views using high‐speed cameras at 600 and 240 Hz, respectively, under stroboscopic LED illumination. Additional high‐speed recordings at higher frame rates were also obtained during preliminary trials when required. A metric grid was used for spatial calibration, and prey items were presented close to the mouth with a fine thread to control their position while minimising disturbance.

Additional details on the experimental equipment, measured variables, device precision and recording procedure are provided in the Supporting Information Material and shown in Figure 2.

**FIGURE 2:**
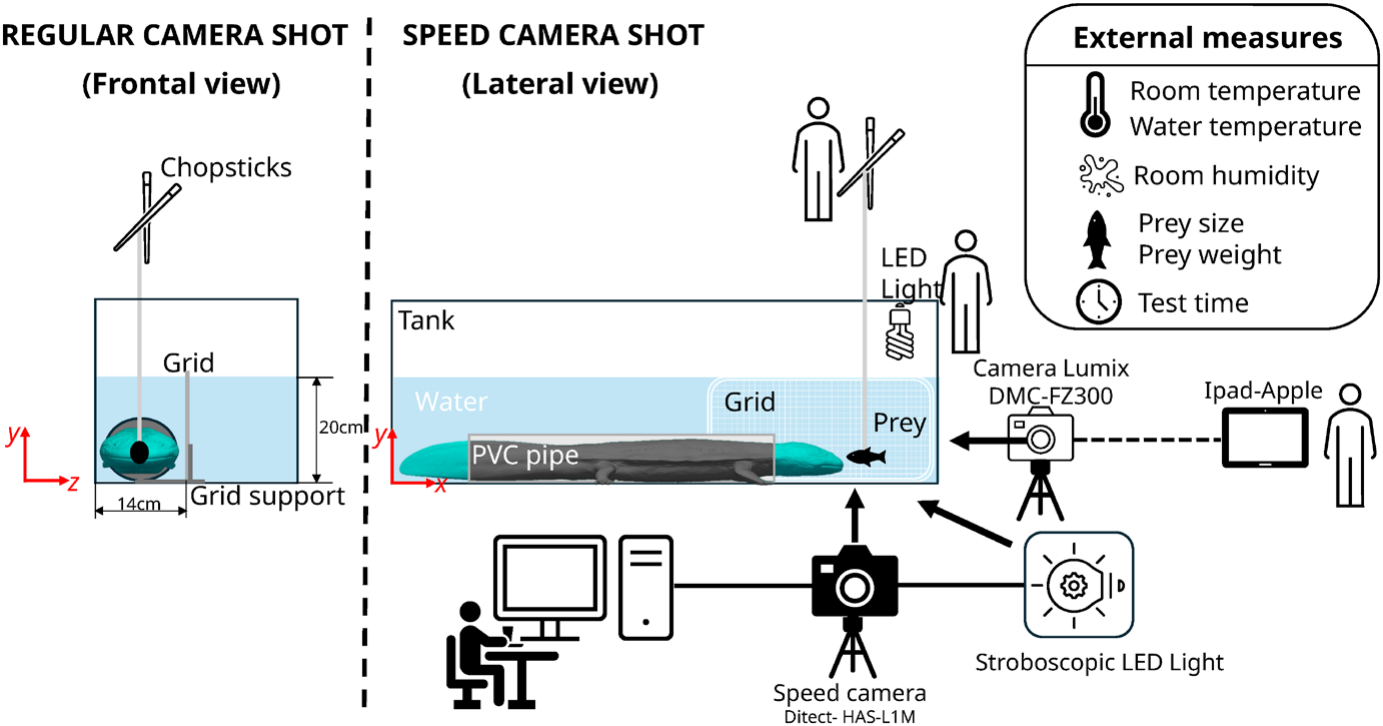
Fully assembled final experimental setup for suction feeding trials.

### 2.5 Suction‐feeding trials

The studied *Andrias* specimens were recorded during individual suction‐feeding trials. Additional details of the recording procedure are provided in the Supporting Information Material. Prey items consisted of standardised fish fragments, prepared from whole fish and measured for length and mass prior to each trial, and were presented using a fine thread with a calibrated knot designed to release the prey during suction. The initial trial for each specimen was performed using a standardised prey item of approximately 1 g in mass and 1 cm in length to provide a comparable baseline among individuals. In subsequent trials, prey size or mass could be adjusted according to the feeding response and behavioural responsiveness of each specimen. Of the six individuals represented in the video‐based dataset, five had a representative event under the standardised 1 g and 1 cm prey condition and were retained for the direct inter‐individual comparison. This procedure maintained comparability among specimens while retaining biologically valid feeding events.

After applying the predefined quality criteria, 20 valid feeding events from six individuals were retained for subsequent analyses (Figure 3). Because not all anatomical landmarks were clearly visible in every recording, sample size differed among analyses: prey‐motion analyses used all valid events, whereas detailed cranial, mandibular and hyobranchial tracking was restricted to the subset of events in which these structures were clearly visible.

**FIGURE 3:**
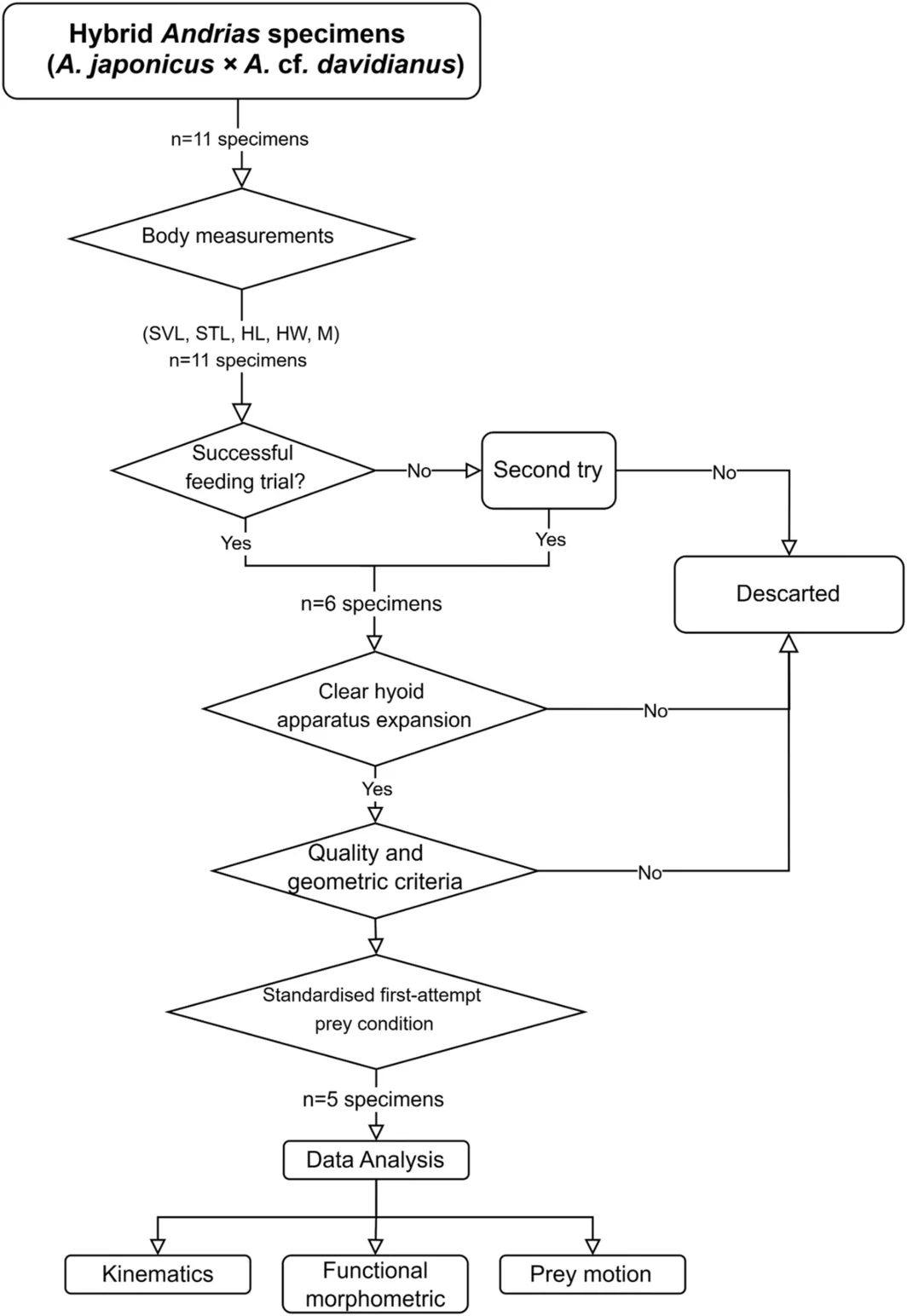
Experimental design and data selection workflow for suction‐feeding trials in hybrid *Andrias* specimens. All morphometric measurements were obtained from all 11 specimens; reductions in sample size apply only to video‐based kinematic and prey motion analyses. The *n* = 5 subset refers specifically to the standardised inter‐individual comparison, whereas the broader video‐based dataset comprised 20 valid feeding events from six specimens.

All individuals were tested under the same protocol, and no treatment groups were defined. Feeding trials were conducted individually, and the order of testing was not determined by specimen size, body condition or expected feeding performance. Environmental conditions, including water temperature, were monitored throughout all trials.

During recording, feeding behaviour had to be monitored directly. To reduce potential observer bias, subsequent kinematic processing and data extraction were conducted without reference to specimen identity or expected functional performance.

### 2.6 Data analysis

Data analysis was exploratory in nature due to the limited number of usable feeding events, an approach commonly adopted in functional and biomechanical studies of suction feeding with limited sample sizes (Heiss et al., 2013; Lauder, 1982; Schwarz et al., 2023). Kinematic variables were calculated at the event level, and descriptive statistics were reported across all valid feeding events. Relationships between peak prey velocity and specimen‐level variables, including BCI, cranial proportions and attack geometry, were examined using specimen‐level summary values to reduce pseudoreplication. Given the limited sample size, these relationships were interpreted descriptively, and no formal inferential statistical tests were applied. For kinematic analysis, the suction‐feeding event was divided into five operational phases: start of mouth opening (SO), hyoid expansion (HE), maximum mouth opening (MMO), mouth closure (C) and gap/slit phase (G) (Figure 4). These phases were used as temporal references to align skull, lower jaw, hyobranchial and prey‐motion data across feeding events.

**FIGURE 4:**
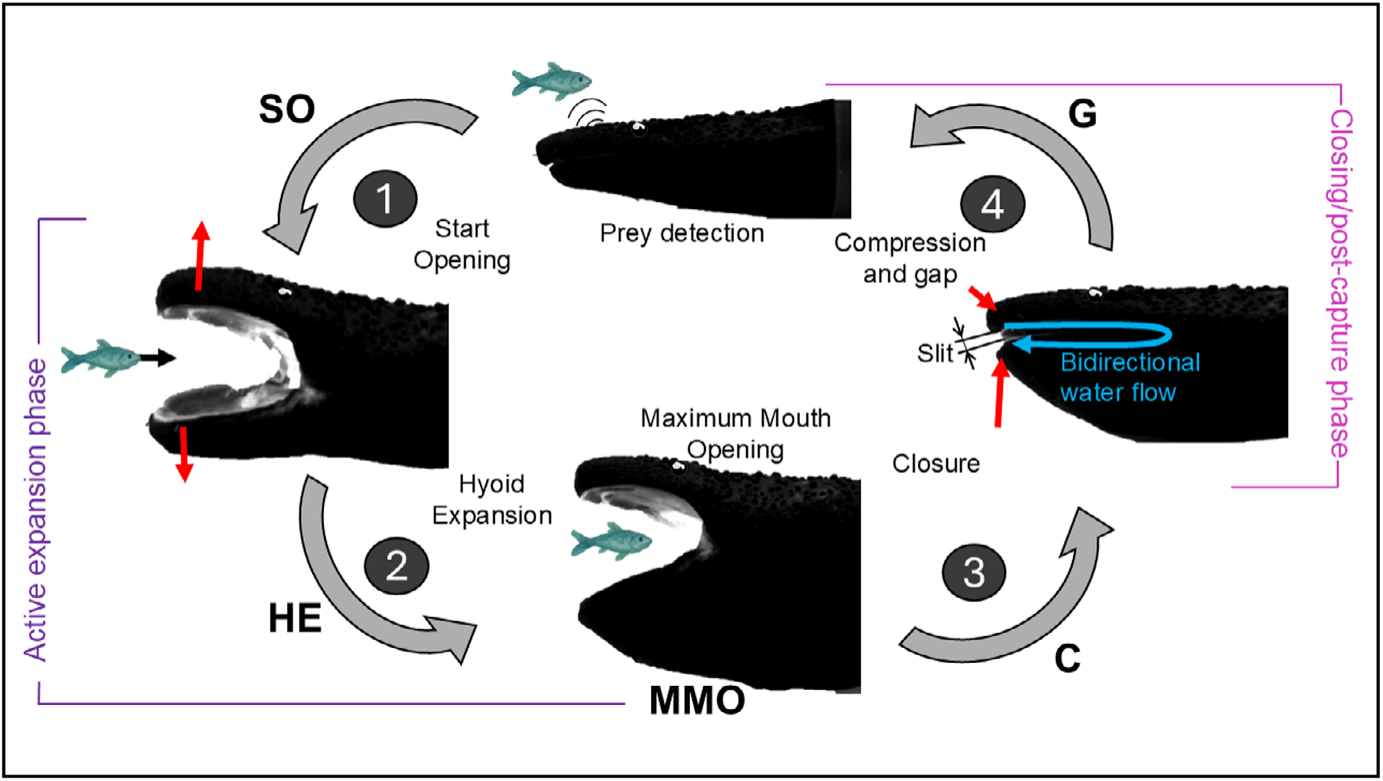
Suction feeding cycle in *Andrias*, based on Kleinteich et al. (2014). Abbreviations indicate key kinematic events within the cycle: C, mouth closure; G, gap/slit formation; HE, hyoid expansion; MMO, maximum mouth opening; SO, start of mouth opening.

This operational phase division follows the functional sequence of suction feeding in *Andrias*, in which mandibular opening and hyobranchial expansion increase the volume of the oropharyngeal cavity and generate inward water flow. In this framework, the active expansion phase extends from SO to MMO and includes HE, whereas the closing/post‐capture phase extends from MMO through C and G. This framework was used only to organise the kinematic data and to compare the timing of prey motion, mandibular movement and hyoid displacement among feeding events.

### 2.7 Kinematic data post‐processing

The experiments were conducted with all available specimens; however, not all produced usable recordings. Some specimens did not feed during the trials, and in other cases, the videos did not meet the required quality standards (Figure 3). Ultimately, 20 valid feeding events from six different specimens were retained for analysis. These events included recordings suitable for prey‐motion analysis, while a smaller subset was suitable for detailed cranial, mandibular and hyobranchial tracking because these anatomical landmarks were not equally visible in all videos (Table 1). These videos met predefined criteria such as symmetry in the animal's position, postural stability, proper alignment with the reference grid, homogeneous illumination and absence of reflections on the tank walls.

**TABLE 1:** Morphometric data and number of valid suction‐feeding events for the six hybrid *Andrias* specimens included in the video‐based analyses. The variable *n* indicates the number of valid trials.

Specimen	STL (cm)	SVL (cm)	HL (cm)	HW (cm)	M (kg)	HL /SVL	HW/HL	BCI (kg/m^3^)	Ontogenetic stage	*n*
1	76	49	11	10	1.70	0.22	0.91	14.45	Adult	3
5	76	52	11.5	11.5	2.54	0.22	1	18.06	Adult	4
6	74.6	47.1	12.6	12.7	2.50	0.27	1.01	23.93	Adult	5
7	72.5	42	11.5	12	2.54	0.27	1.04	34.28	Adult	1
8	88	59	12.5	12.5	2.74	0.21	1	13.34	Adult	6

All available hybrid specimens were included at the beginning of the study. For descriptive kinematic analyses, the experimental unit was the suction‐feeding event, and multiple valid events per specimen were retained when available. For associations involving specimen‐level variables, such as BCI and cranial proportions, specimen‐level summary values were used to reduce pseudoreplication.

All inclusion and exclusion criteria were defined prior to the start of the experiments and data collection. Exclusions were based solely on functional, geometric and recording‐quality criteria required to properly analyse the different phases of the suction‐feeding cycle (Figure 4).

Post‐processing was performed using the Tracker software (v6.3.3; Open Source Physics; https://physlets.org/tracker/; [RRID:SCR_027845]). First, a Cartesian coordinate system (X, Y) was established and corrected frame by frame according to the selected anatomical reference point. Anatomical landmarks were then digitised in each frame, including the skull, mandible, hyoid and prey (Figure 5). This reference point was used to quantify the horizontal displacement associated with frontal head movements during suction feeding. From the temporal series of *x* and *y* positions, linear velocity components were calculated using finite‐difference approximations: forward and backward differences at the boundaries of the signal, and central differences at internal points, in order to minimise truncation error (Chapra & Canale, 2015). Prey velocity ($V_{\text{prey}}$) was expressed as the magnitude of the two‐dimensional velocity vector, calculated as $V_{\text{prey}}=\sqrt{v_{x}^{2}+v_{y}^{2}}$. Signed tangential prey acceleration was subsequently calculated as the temporal derivative of the smoothed prey velocity, $a_{\text{tangential},\text{prey}}=dV_{\text{prey}}/\mathrm{dt}$, using central differences at internal points, such that positive and negative values indicate increases and decreases in prey velocity, respectively. For comparison, the magnitude of the two‐dimensional prey acceleration vector $a_{\text{magnitude},\text{prey}}=\sqrt{a_{x}^{2}+a_{y}^{2}}$ was also calculated. Hyoid displacement was calculated as the two‐dimensional displacement of the tracked hyoid reference point relative to its initial position.

**FIGURE 5:**
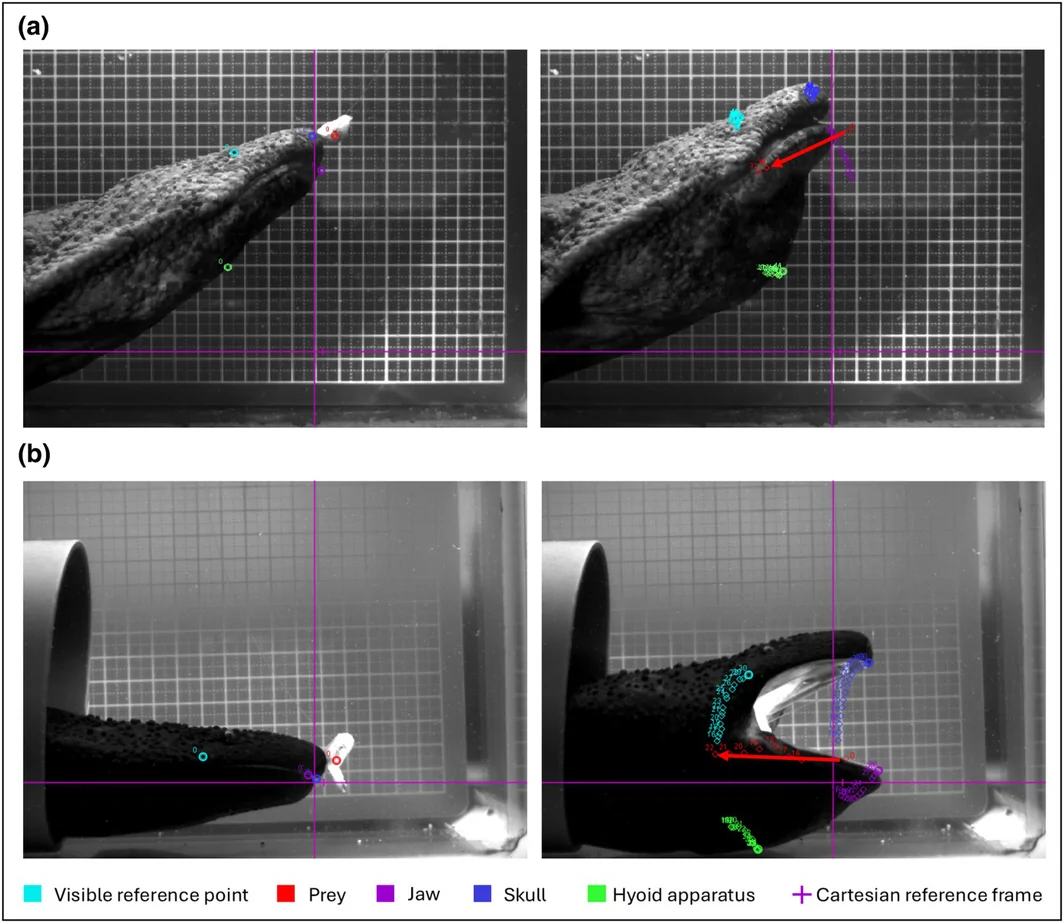
Definition of an externally visible reference point and digital tracking of skull, jaw and hyoid movements. Examples of reference points are shown: (a) specimen 6, using the eye as the reference point, illustrating displacement up to mandibular closure, and (b) specimen 1, using a scale adjacent to the mouth as the reference point, illustrating displacement up to maximum mouth opening (MMO).

All digitised kinematic data were processed to reduce measurement noise, with signal smoothing applied particularly for acceleration calculations. For this purpose, the Savitzky–Golay filter implemented in the SciPy library (scipy.signal.savgol_filter) was applied using custom Python scripts (Python; [RRID:SCR_008394]) (Savitzky & Golay, 1964). Full details of the error treatment and smoothing procedures are provided in the Supporting Information Material.

### 2.8 Postural measurements

The attack angle (θ) was quantified from lateral‐view high‐speed videos using Tracker software (Open Source Physics). A horizontal reference axis was defined based on the tank geometry. The orientation of the head–prey system was estimated by a straight line connecting the geometric midpoint of the prey and the midline of the head in lateral view. The angle between this line and the horizontal reference was measured at the onset of prey acceleration. Given the postural variability of the animals and the two‐dimensional projection of the recordings, θ is interpreted as an approximate descriptor of attack orientation rather than a precise anatomical measurement.

## 3 RESULTS: FUNCTIONAL OUTCOMES OF THE SUCTION‐FEEDING APPARATUS

The outcome measures in this study are based on kinematic and functional variables obtained from high‐speed video recordings of suction‐feeding events. These include cranial, mandibular and hyoid kinematics, prey motion and functional morphometric descriptors. Analyses were restricted to specimens with analysable suction‐feeding events to allow interpretable morphology–performance relationships.

Descriptive statistics (including ranges and specimen values) were used to summarise all measured variables. All events that met predefined quality criteria were included in the results.

### 3.1 Morphometric variation within the hybrid sample

Morphometric ratios and BCI varied among the analysed hybrid specimens (Figure 6 and Table 1). The relationship between HL/SVL and the BCI showed that specimens 6 and 7 had the highest relative head‐length values and among the highest BCI values in the dataset (Figure 6a). In contrast, specimens 1 and 8 showed lower HL/SVL and lower BCI values.

**FIGURE 6:**
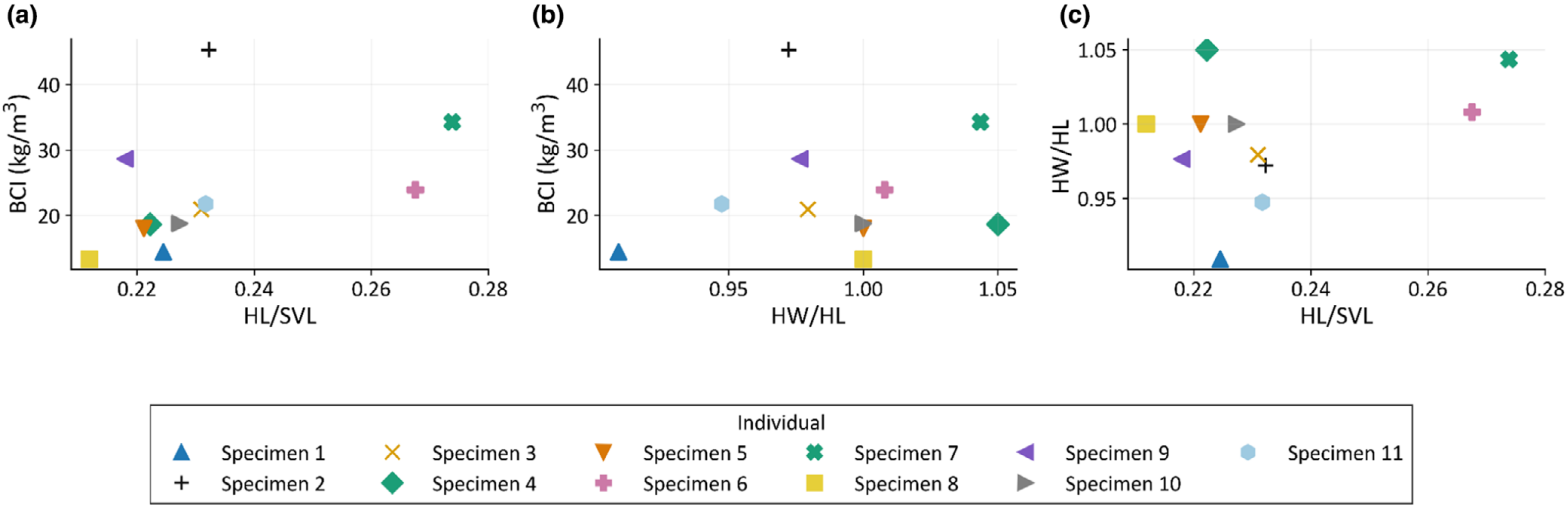
Morphometric variation within the hybrid *Andrias* sample. Relationships between (a) HL/SVL and BCI, (b) HW/HL and BCI and (c) HL/SVL and HW/HL.

A similar trend is observed in the relationship between HW/HL and BCI. Specimens with relatively wider heads also tended to show higher BCI values (Figure 6b and Table 1). Specimen 7 showed the highest HW/HL ratio, while specimen 1 showed one of the lowest relative head‐width values. Overall, HW/HL varied within the hybrid sample, indicating differences in cranial proportions among specimens.

The comparison between HL/SVL and HW/HL further highlights variation in cranial proportions within the hybrid sample (Figure 6c and Table 1). Those with relatively long heads, such as specimens 6 and 7, also tend to have wider heads. In contrast, specimens with lower values for both ratios, including specimens 1 and 8, showed lower cranial proportion values.

Specimens 1 and 8 showed consistently low BCI values relative to the remaining specimens. Specimen 1 also showed the lowest HW/HL value, whereas specimen 8, despite being the longest specimen in the group, showed a relatively low HL/SVL value and one of the lowest BCI values. These patterns describe morphometric and body‐condition variation within the hybrid sample, but they do not allow direct inference of nutritional history or developmental condition (Figure 6).

### 3.2 Kinematic sequence and inferred suction‐flow generation

Kinematic analysis of the high‐speed videos confirmed the expected suction‐feeding sequence (SO → HE → MMO → C → G) across the analysed feeding events. Because flow was not measured directly, flow generation is inferred here from coordinated cranial, mandibular, hyobranchial and prey‐motion patterns. Frame‐by‐frame tracking provided positional trajectories for the skull, lower jaw and hyobranchial apparatus and prey, enabling the calculation of instantaneous velocities and accelerations. These data describe variation in the timing and magnitude of movements among specimens and events. Trial‐level prey‐motion profiles for all valid feeding events are provided in Figure S1. The magnitude of the two‐dimensional prey acceleration vector for the representative feeding events is presented in Figure S2. This analysis focuses on the prey‐capture component of the feeding sequence and does not address subsequent intraoral transport, processing or swallowing.

### 3.3 Prey motion as the functional outcome of suction

Tracking the prey provided a direct measure of the suction‐feeding event. While the kinematics of the skull, lower jaw and hyoid describe the movements responsible for generating the flow, prey displacement reflects the outcome of these movements within the fluid. Frame‐by‐frame tracking allowed extraction of the prey's positional trajectory, as well as its velocities and accelerations, allowing prey displacement, velocity and acceleration to be compared among specimens and events. Of the six individuals included in the video‐based analyses, five could be directly compared under approximately standardised prey conditions, using prey fragments of about 1 cm in length and 1 g in mass. For these five specimens, representative trials were compared in terms of prey velocity, signed tangential acceleration and hyoid displacement (Figure 7a–c). Peak prey velocity ranged from 1.31 to 2.19 m/s across the five representative trials, with values of 2.19 m/s for specimen 1, 1.67 m/s for specimen 8, 1.40 m/s for specimen 6 and 1.31 m/s for specimens 5 and 7 (Figure 7a).

**FIGURE 7:**
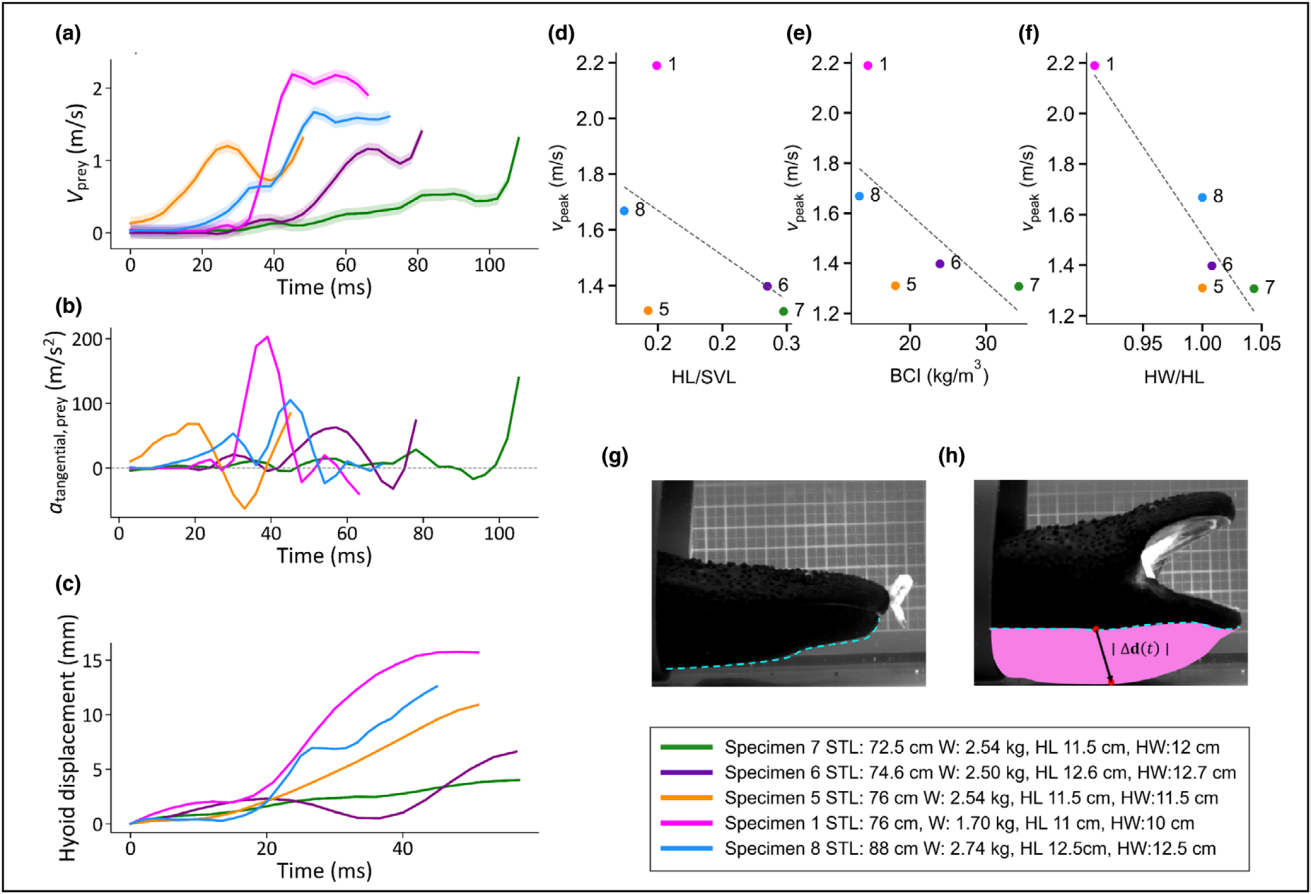
Prey kinematics and hyoid displacement up to the instant of maximum mouth opening (MMO) during representative successful suction‐feeding events in hybrid *Andrias* specimens. All events shown correspond to the 1 g prey condition, which was used to standardise inter‐individual comparisons. (a) Prey velocity magnitude derived from positional data; (b) signed tangential prey acceleration, calculated from the smoothed prey‐velocity curves, where positive and negative values indicate increases and decreases in velocity magnitude, respectively; (c) hyoid displacement curves during the active expansion phase; (d) peak prey velocity in relation to head length normalised by snout–vent length; (e) peak prey velocity in relation to body condition index (BCI); (f) peak prey velocity in relation to relative head width (HW/HL); (g) pre‐SO hyoid reference position; and (h) hyoid position at MMO and displacement vector of a reference point. Shaded bands in panel (a) indicate estimated tracking uncertainty, whereas dashed lines in panels (d–f) indicate descriptive trends only.

Signed tangential prey acceleration ranged from −62.88 to 202.98 m/s^2^ across the representative trials. Specimen 1 showed the highest positive acceleration (202.98 m/s^2^), whereas specimen 5 showed the strongest deceleration (−62.88 m/s^2^). Negative acceleration values were observed in all five specimens, indicating intervals of decreasing prey velocity magnitude (Figure 7b).

Maximum hyoid displacement varied from 3.99 to 15.76 mm across the five representative trials, with specimen 1 showing the greatest displacement (15.76 mm), followed by specimens 8 (12.59 mm), 5 (10.91 mm), 6 (6.61 mm) and 7 (3.99 mm) (Figure 7c).

## 4 DISCUSSION

### 4.1 Overview of the results

This study examined suction feeding in hybrids of the genus *Andrias* by combining morphometric data with kinematic analyses of prey motion and hyobranchial apparatus expansion. The results show that hybrid specimens retain a suction‐feeding pattern consistent with that described for non‐hybrid *Andrias*, falling within the known functional range of the genus. Although the overall sequence of the suction feeding event was consistent among specimens and closely followed the feeding cycle described for the *Andrias* clade (Figure 4), variation was observed in the intensity and execution of the feeding events across trials. These differences were most evident during the active expansion phase of the cycle when the largest changes in prey velocity and acceleration occurred.

### 4.2 Do hybrids fall within the non‐hybrid functional range?

A recent geometric morphometric study has shown that hybrid *Andrias* specimens exhibit an overall intermediate cranial morphology, with a tendency toward proportionally wider mouths than either parental species (Hara et al., 2025, and references therein). This trait may have functional implications during prey capture, particularly given that *Andrias* does not rely exclusively on strictly axial suction but can incorporate lateral cranial deviations and lateral biting manoeuvres (Matsumoto et al., 2024).

Despite these morphological features, the hybrids analysed here displayed a suction feeding pattern comparable to that described for non‐hybrid *Andrias*, including *A. davidianus and A. japonicus* (Heiss et al., 2013; Matsumoto et al., 2024). The basic sequence of mandibular opening (SO), hyoid expansion (HE) and prey capture was conserved across feeding events. This pattern indicates that hybridisation does not appear to disrupt the fundamental biomechanical principles of suction feeding and that the functional architecture of the system is highly conserved.

However, these comparisons are based on previously published datasets for *A. davidianus* and *A. japonicus* (Heiss et al., 2013; Matsumoto et al., 2024) obtained under different experimental conditions and therefore should be interpreted as a qualitative functional comparison rather than a direct experimental test between hybrid and non‐hybrid individuals. Thus, published data for non‐hybrid *A. davidianus* and *A. japonicus* are used here as a functional baseline rather than as direct experimental controls.

Within the limits of the present dataset, the observed variability appears to be more closely associated with specimen‐level factors such as body condition, body mass and posture during capture than with any detectable hybrid‐specific feeding pattern.

### 4.3 How do morphology and body condition relate to performance?

A relationship between body condition and inferred suction performance was observed when maximum prey velocity was analysed as a function of the BCI (Figure 7e). A descriptive negative trend was apparent, with individuals showing lower BCI generally tending to reach higher peak prey velocities. Given the limited sample size, this pattern is treated as exploratory and should not be interpreted as evidence of a causal relationship. Here, BCI is used only as a descriptive proxy for individual body condition, whereas short‐term feeding motivation may also have contributed to event‐level variation in suction performance.

A similar source of event‐level variation may be related to trial order. In several specimens, the first successful feeding attempt appeared to produce the highest prey‐motion response, particularly in terms of peak prey velocity and movement speed (see Figure S1 for specimen‐level trial results). This pattern should be interpreted cautiously, because trial order was not experimentally controlled as an independent variable. However, it is consistent with the idea that prey‐capture kinematics can be modulated by short‐term feeding motivation or satiation state, as reported in other suction‐feeding vertebrates (Sass & Motta, 2002). In the present study, this observation is therefore treated as an exploratory indication that initial behavioural responsiveness may contribute to variation in suction‐feeding performance.

Specimen 5 differed from the general descriptive BCI–velocity pattern, combining a comparatively low BCI with a relatively low peak prey velocity. Given the limited sample size, we retained this specimen and interpreted it from a functional rather than statistical perspective. Its behaviour is discussed in more detail in relation to prey positioning and head orientation.

Morphometric patterns were also reflected in the relationship between head proportions and maximum prey velocity (Figure 7d–f). In particular, Figure 7d relates maximum prey velocity to the head length to snout–vent length ratio (HL/SVL), which reflects relative head elongation rather than body or head width. A general negative trend was observed, indicating that specimens with proportionally longer heads relative to body length tended to exhibit lower peak velocities.

This trend is consistent with the exploratory relationships identified across morphometric variables (Figure 7d–f). It was most evident in specimen 7, which combined greater relative head width with one of the lowest peak prey velocities.

By contrast, specimens with lower relative head‐length values reached relatively high peak prey velocities, suggesting that suction performance cannot be explained by a single morphometric axis. This may reflect aspects of cranial geometry not fully captured by simple proportional indices, differences in hyoid expansion dynamics, reduced inertial constraints or a combination of these factors. However, these interpretations remain tentative and require further validation with larger sample sizes.

These patterns indicate that cranial morphology alone does not fully explain variation in inferred suction performance, because specimens with comparatively low HL/SVL and HW/HL values, such as specimen 1, can still achieve high inferred suction performance. This suggests that execution‐related variables, such as posture, attack geometry and timing of cranial expansion, may interact with cranial proportions, contributing substantially to performance differences among hybrids.

Taken together, these results indicate that morphometry appears to influence suction feeding performance but does not determine it in isolation. Its effects likely interact with body condition, prey position, posture during capture and other event‐level behavioural factors that were not directly quantified in this study.

The negative trend between relative head width and peak prey velocity may reflect an association between cranial proportions and inferred suction performance (Figure 7f), while the hyoid expansion highlights the key, conserved role of the hyobranchial apparatus in salamander suction.

In this context, the analysis of hyobranchial apparatus expansion in lateral view provides additional insight into functional variability among specimens. Displacement curves were referenced to the first tracked hyoid position and time point for each specimen (Figure 7c, g, h), allowing direct comparison of displacement magnitude and temporal progression.

Using this common temporal reference, inter‐individual variation in hyoid displacement was observed. Specimens 1 and 8, both classified as adults, exhibited rapid and large‐amplitude hyoid expansion, whereas the other representative individuals showed slower or more limited displacement (Figure 7c). Notably, specimen 1 displayed the largest hyoid displacement despite having relatively low HL/SVL and HW/HL values, suggesting that individual kinematics and capture behaviour may contribute substantially to suction performance.

These results support the idea that feeding effectiveness depends not only on external morphology but also on functional and behavioural factors acting during the active expansion phase of the feeding cycle.

### 4.4 Does suction performance vary with event‐level behaviour?

Signed tangential prey acceleration showed a recurrent, although variably expressed, bimodal pattern in several representative feeding events (Figure 7a, b). An early phase of positive acceleration was followed in several specimens by a second increase in prey velocity, although the relative magnitude and timing of these phases varied among individuals. Negative acceleration values occurred between or after these phases, reflecting temporary decreases in prey velocity magnitude. Specimen 1 showed the highest positive tangential acceleration, whereas specimen 5 showed the strongest deceleration.

Because the specimens considered in this kinematic comparison were all classified as adults and occupied a relatively restricted adult size range, this variation cannot be attributed to ontogenetic stage alone. Although the full hybrid sample included both subadult and adult individuals, a broader and more evenly distributed sample across size classes would be required to test whether body size or ontogeny systematically affects prey acceleration during suction feeding. Therefore, the observed differences are interpreted here as variation among individuals and among feeding events, likely influenced by prey position, attack configuration, hyoid kinematics and head orientation during capture.

Recurrent asymmetries were observed in both mandibular opening and hyoid displacement. In cryptobranchids, such asymmetries have been interpreted as a functional feature of the group rather than a pathological deviation (Cundall et al., 1987; Matsumoto et al., 2024). These asymmetries may facilitate capture when the prey is not aligned with the sagittal axis, or may reflect local differences in muscular activation.

Their consistent occurrence in the hybrid specimens analysed here suggests that suction feeding in hybrid *Andrias* also operates as a directional and adaptable manoeuvre, rather than as a strictly symmetrical process (Figure 8c).

**FIGURE 8:**
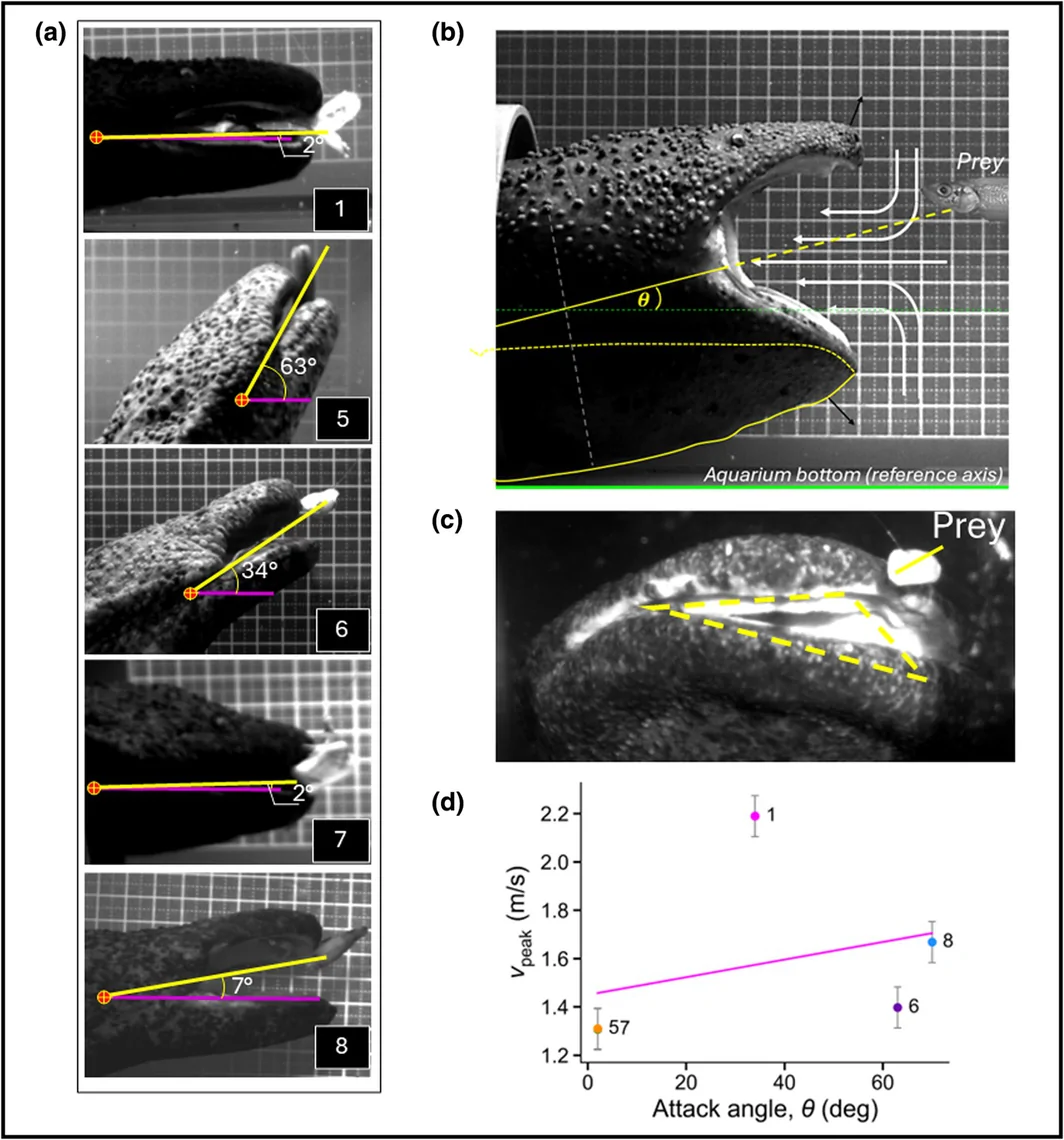
(a) Attack angle (θ) for each specimen; (b) geometric definition of the attack angle (θ) during suction feeding in a hybrid *Andrias*; (c) frontal view of an asymmetric suction event in specimen 6 (5000 Hz); (d) peak prey velocity as a function of attack angle (θ). In panel (d), the line indicates a descriptive trend only, while error bars represent estimated tracking uncertainty.

All successful captures shared a common spatial feature: prey were located extremely close to the oral margin at the onset of suction. Regardless of prey size, orientation or body inclination, capture was observed only when prey were within approximately 5 mm of the mouth at the initiation of mandibular expansion. This restricted capture distance is consistent with a spatial constraint of suction feeding, in which the effectiveness of prey entrainment decreases rapidly with distance from the mouth opening.

Behaviourally, salamanders adopted a clear ambush strategy, remaining immobile for extended periods and waiting for prey to approach actively, often until contacting the snout (Figure 8a). This suggests that suction initiation may be associated with short prey distances, potentially maximising success in a single feeding attempt.

Head orientation varied among specimens, demonstrating that suction feeding does not require a horizontal posture (Figure 8a). While some specimens captured prey at near‐zero attack angles, others exhibited moderate to pronounced cranial inclinations. This interspecimen variation is reflected quantitatively in the relationship between attack angle and peak prey velocity (Figure 8b, d) although no clear relationship can be inferred from the limited sample.

Within this broader pattern, specimen 5 represents the most extreme case, capturing prey at an angle close to 63°. This observation shows that successful suction feeding can occur at a pronounced head orientation relative to the trunk. Despite this different orientation, specimen 5 was retained as a biologically informative example of functional variation rather than treated as a recording artefact. Specifically, the recording for this specimen met all predefined quality criteria: the video quality was high, the full feeding cycle was captured, and complete hyoid expansion was visible. Thus, excluding this observation would have removed a biologically meaningful example of functional variability.

All specimens exhibited anterior head displacement during suction, ranging from 1 to 3 cm depending on attack angle. This forward motion was consistently observed and appears to be an integral component of the suction feeding cycle. Among the analysed valid events, all suction events resulted in successful prey capture once suction was initiated.

However, this observed success rate under captive experimental conditions should not be interpreted as representative of capture success in natural environments, where factors such as surrounding water movement and prey escape responses may alter feeding outcomes.

### 4.5 Ecological and evolutionary implications

The apparent contribution of body condition, morphometry and spatial configuration to performance suggests that feeding in *Andrias* is a flexible and context‐dependent system. Such variability may be advantageous in environments where prey availability and spatial conditions change, allowing individuals to adjust their feeding behaviour accordingly. The conservation of feeding performance in hybrids supports the idea that the core biomechanical architecture of this feeding system is robust to hybridisation, at least within the functional range examined in this study. However, this interpretation is based on qualitative comparisons with previously published data and should not be taken as evidence of complete functional equivalence with parental species.

From a conservation perspective, the present results highlight that feeding performance in hybrids may vary with individual condition and morphology within the hybrid sample. This suggests that functional traits relevant to survival, such as prey capture efficiency, cannot be inferred solely from genetic background without considering phenotypic and behavioural variability.

While these findings provide a first functional perspective on hybrid *Andrias*, further studies integrating ecological data, population‐level analyses and direct comparisons with non‐hybrid individuals are required to fully assess the implications of hybridisation for conservation and ecosystem dynamics.

### 4.6 Study limitations

The main limitation of this study was the difficulty of recording complete suction feeding events, particularly those with clearly resolved hyoid expansion. *Andrias* salamanders are highly sensitive to disturbance and may suppress feeding under altered conditions, which limited the number of usable events and constrained sample size.

In addition, analyses focused exclusively on the prey‐capture component of the feeding sequence and did not include subsequent intraoral transport, processing or swallowing. Captive conditions and the use of a single prey type may have influenced behaviour. Finally, the absence of direct pressure measurements and three‐dimensional flow reconstructions limits a full characterisation of hydrodynamic performance.

These limitations define the next experimental step: direct comparisons between hybrid and non‐hybrid individuals under controlled prey size, feeding history and direct flow and pressure measurement conditions.

## 5 CONCLUSIONS

This study shows that suction feeding in hybrid *Andrias* retains the fundamental biomechanical organisation described for cryptobranchids. The sequence of mandibular opening, hyoid expansion and prey acceleration was consistently observed across valid events, indicating that hybridisation did not appear to disrupt the core suction‐feeding pattern within the functional range examined here. Successful prey capture across different postures and attack angles further suggests that this feeding mechanism is mechanically robust and flexible in execution.

Differences in suction performance appeared to vary with individual condition, morphometry and the spatial configuration of the feeding event, but these associations should be interpreted as exploratory rather than causal. In particular, variation among events suggests that performance is modulated by the interaction between morphological and behavioural factors, rather than being determined by a single variable.

Lower BCI values coincided with higher peak prey velocities in some events, but this pattern should not be interpreted as a direct effect of fasting or feeding motivation, because individual feeding histories were not fully controlled prior to the final 24 h food‐withholding period. Similarly, attack angle appeared to be related to prey‐capture execution, although its relationship with inferred suction performance remains exploratory. Even at higher attack angles, suction remained sufficient to capture prey located at very short distances.

Overall, these results indicate that suction feeding in hybrid *Andrias* is functionally consistent with the pattern previously described for the genus, while showing event‐level variation in execution. These findings should be interpreted as exploratory and provide a basis for future studies incorporating larger sample sizes, controlled feeding histories and direct comparisons with non‐hybrid individuals or populations.

More broadly, these findings support the view that suction feeding in *Andrias* is a conserved but flexible form–function system, in which morphology, prey position and behavioural execution jointly shape capture performance.

## AUTHOR CONTRIBUTIONS

M. López‐Pachón conceived and designed the study, conducted the experiments, performed data post‐processing and analyses and drafted the manuscript. J. Fortuny contributed to biological interpretation and supported data analysis and manuscript writing. S. Hayashi facilitated access to the specimens and contributed to experimental support and data acquisition. J. Marcé‐Nogué contributed to study design, data analysis, supervision and manuscript writing. All authors critically reviewed the manuscript.

## CONFLICT OF INTEREST STATEMENT

The authors declare no competing interests.

## Supporting information


**Table S1.** Morphometric data for hybrid Andrias specimens.
**Table S2**. Experimental equipment and estimated uncertainties.
**Figure S1**. Trial‐level prey‐motion profiles for all valid feeding events in each analysed specimen. Curves show normalised X displacement, Y displacement, prey velocity magnitude and signed tangential prey acceleration. For specimens with multiple feeding trials, labels include the inferred display order in parentheses. The 1 g and 1 cm prey condition was assigned as T1 because this was the known first trial. Specimens with only one valid trial are shown without an order label. In the panel labels, ‘Speed’ refers to prey velocity magnitude and ‘Acceleration’ refers to signed tangential prey acceleration.
**Figure S2**. Magnitude of the two‐dimensional prey acceleration vector during representative suction‐feeding events in hybrid *Andrias* specimens. Prey acceleration magnitude was calculated as $a_{\text{magnitude},\text{prey}}=\sqrt{a_{x}^{2}+a_{y}^{2}}$, where $a_{x}$and $a_{y}$represent the horizontal and vertical acceleration components, respectively. Unlike the signed tangential acceleration shown in Figure 7(b), this quantity is necessarily non‐negative and reflects changes in both the Prey velocity magnitude and direction of prey motion. Curves correspond to the same representative specimens shown in Figure 7.

## Data Availability

The high‐speed videos supporting this study (lateral‐view recordings of suction‐feeding events) have been deposited in Zenodo (DOI: 10.5281/zenodo.18153583). The repository is currently under restricted access and will be made publicly available upon publication of this manuscript.
