# Supplementary material for: Suction‐feeding performance in invasive alien giant salamanders within the functional framework of Andrias
Source: J Anat. 2026 Oct 24:10.1111/joa.70262. Online ahead of print. doi: 10.1111/joa.70262 (PMC13695036; doi:10.1111/joa.70262)
Supplement: Supplementary file 1 [file JOA-9999-0-s001.zip › joa70262-sup-0001-Supinfo01.docx]

**Supplementary information**

**Suction-feeding performance in invasive alien giant salamanders within the functional framework of *Andrias***

M. López-Pachón^1,2^, S. Hayashi^2,3^, J. Fortuny^4^ and J. Marcé-Nogué^1^

**Running title:** Suction feeding in hybrid *Andrias*

**Correspondence:** Jordi Marcé-Nogué, Email: jordi.marce@urv.cat

¹Universitat Rovira i Virgili, Department d'Enginyeria Mecànica, Campus Sescelades, Edifici E4, Avinguda Països Catalans 26, 43007, Tarragona, Spain

^2^Okayama University of Science, Okayama, Japan

^3^The University of Osaka, Osaka, Japan

^4^Institut Català de Paleontologia Miquel Crusafont (ICP-CERCA), Universitat Autònoma de Barcelona, Edifici ICTA-ICP, c/ de les Columnes s/n, Campus de la UAB, 08193 Cerdanyola del Vallès, Barcelona, Catalonia, Spain.

# 1 TRIAL DETAILS

## 1.1 Experimental Setup

Each specimen was placed in a 45×90×45 cm glass aquarium, which provided an adequate water depth and a stable field of view for lateral filming (**Figure 2**). To obtain recordings that were as lateral, centred and symmetrical as possible, the specimen was positioned inside a PVC tube placed within the aquarium, which restricted its lateral movement and facilitated proper alignment with the recording camera.

Outside the experimental trials, specimens were fed primarily with fish similar to those used during the feeding experiments, such as Japanese smelt (*Hypomesus nipponensis*) and Japanese jack mackerel (*Trachurus japonicus*) (**Figure 1b-c**), with occasional supplementation depending on specimen needs and feeding behaviour. The prey was presented using a nylon thread, held and manoeuvred with chopsticks, allowing precise control of its position and enabling the simulation of natural prey-like movements around the animal’s head.

The main recording system consisted of a DITECT HAS-L1M high-speed lateral camera (DITECT Corporation, 2022) capable of frame rates up to 18,000 frames per second, which was used to capture the details of mandibular opening, hyoid displacement, and prey trajectory during suction. Illumination was provided by a stroboscopic LED lighting system, essential for minimising motion blur and ensuring optimal contrast during high-frequency recordings. A 1×1 cm metric grid was placed behind the PVC pipe to allow spatial calibration of the lateral view. Prior to each trial, environmental variables (laboratory temperature, water temperature, humidity, and time) were recorded (**Figure 2**).

## 1.2 Recording procedure

Frontal recordings were generally obtained at 240 Hz, whereas standard lateral recordings were captured at 600 Hz. These frame rates provided an effective compromise between file size, video duration, and image clarity under strobe illumination. Additional high-speed recordings at higher frame rates were also obtained during preliminary trials when required.

Each experiment required four operators: one per camera, one handling the prey, and one controlling the synchronisation LED and temporal labelling (**Figure 2**). The prey was slowly introduced into the aquarium and positioned near the salamander’s head to avoid premature stimulation, aiming for an initial separation of approximately 2 cm from the snout when possible. The synchronisation operator then displayed an identification card, performed a countdown, and gave the command “action.”

Both cameras were then activated, the prey operator applied controlled movements to mimic swimming, and the LED operator triggered the on/off pulse for temporal alignment. After the suction-feeding event, both recordings were stopped simultaneously, and this procedure was repeated for all trials.

# 2 ERROR TREATMENT AND SIGNAL SMOOTHING

## 2.1 Measurement uncertainty and error propagation

Velocity and acceleration were computed by numerical differentiation with respect to time, and the precision of these estimates depends directly on the accuracy with which point positions were determined in each video frame. Because the number of valid feeding events differed among specimens, the uncertainty analysis focused on random measurement error associated with the digitisation of point positions and on its amplification during numerical differentiation, rather than on biological variability across repeated trials (Winter, 2009). This approach is commonly adopted in biomechanical studies of suction feeding, where obtaining controlled replicates is often impractical (Heiss et al., 2013; Lauder, 1982).

High-speed recordings were obtained with a DITECT HAS-L1M camera operating at 600 fps, yielding a temporal resolution of Δt = 1/600 s, equivalent to 0.00167 s or 1.67 ms. Digitizing precision was estimated at approximately ±0.5 pixels, with a spatial scale of ~0.2 mm per pixel. The resulting positional uncertainty is:

$$\sigma_{x}=0.5\text{ px}\times0.2\text{ mm/px}=0.1\text{ mm}=1\times{10}^{-4}\text{ m}.$$

Velocity was computed using finite differences: forward and backward differences at the boundaries, and central differences for all interior points. The positional uncertainty propagates to the velocity estimate according to:

$$\sigma_{v}\approx\frac{\sqrt{2}\text{ }\sigma_{x}}{\Delta t},$$

which was used to construct uncertainty envelopes based on propagated positional error around each smoothed velocity curve. These envelopes provide a conservative estimate of variation attributable to digitisation noise and aid in assessing the robustness of inter-individual comparisons.

Signed tangential prey acceleration was calculated as the temporal derivative of the smoothed prey velocity magnitude, $a_{tangential,prey}=dV_{\mathrm{prey}}/dt$. Numerical differentiation remains sensitive to tracking noise (Winter, 2009); therefore, tangential acceleration traces were interpreted qualitatively and no uncertainty bands were plotted for acceleration. The magnitude of the two-dimensional acceleration vector, $a_{magnitude,prey}=\sqrt{a_{x}^{2}+a_{y}^{2}}$, was retained separately and is presented in Supplementary **Figure S2**.

## 2.2 Spatial calibration and evaluation of instrumental error

A 1 cm grid was placed behind the salamander and remained visible in all frames of the recordings (**Figure 2**). This grid served simultaneously as the spatial calibration reference and as a tool to evaluate potential systematic errors associated with the recording system. To verify scale accuracy, ten consecutive grid squares were measured along both the X and Y axes (Hatze, 1981). The resulting distances deviated by less than 0.5 per cent from the expected 100 mm, indicating that the spatial calibration remained stable and without appreciable drift throughout the high-speed recordings.

The uniform size of the grid squares across the entire image confirmed that the camera sensor was effectively parallel to the plane of motion, thereby minimising perspective-related distortions. Likewise, the straightness of the horizontal and vertical grid lines indicated no appreciable radial or tangential lens distortion. Such optical deformations typically appear as curvature or displacement of the grid pattern, but in the recordings analysed, all lines remained straight and evenly spaced, showing that the image geometry accurately reflected the physical recording plane.

Before post-processing, all available sequences were inspected, and only those in which the grid exhibited stable and homogeneous calibration across the full field of view were retained. Recordings showing irregularities in scale or any indication of geometric distortion were excluded to prevent the introduction of systematic bias into the kinematic analysis. Consequently, the videos included in the final dataset met predefined geometric quality criteria and provided consistent and reliable positional data. Although the systematic errors associated with the recording apparatus were small compared to the random digitisation error, their identification and control were essential to ensure that overall uncertainty primarily reflected measurement variability rather than instrumental deficiencies ( Zhang, 2000).

## 2.3  Smoothing of kinematic signals

Raw position, velocity, and acceleration traces exhibit high-frequency fluctuations originating from digitisation noise and small visual inconsistencies inherent to high-speed video recordings. To reduce such variability while preserving the temporal structure of the movement, we applied a Savitzky–Golay filter to the derived prey-velocity magnitude and hyoid-displacement series (Savitzky and Golay, 1964). Signed tangential prey acceleration was subsequently calculated from the smoothed prey-velocity magnitude. This filter preserves curvature and peak morphology more effectively than simple moving averages, making it particularly suitable for suction-feeding dynamics, where rapid transitions and brief velocity maxima are biologically meaningful (Challis, 1999).

In this study, we used a 7-point window and a third-order polynomial, a combination that provides strong noise reduction without distorting the timing or magnitude of key kinematic events. The filtered signal at each point is given by:

$$\hat{\boldsymbol{y}}\boldsymbol{(}\boldsymbol{t}_{\boldsymbol{i}}\boldsymbol{)=}\sum_{\boldsymbol{j}\boldsymbol{=-}\boldsymbol{m}}^{\boldsymbol{m}} \boldsymbol{c}_{\boldsymbol{j}}\text{ }\boldsymbol{y}\boldsymbol{(}\boldsymbol{t}_{\boldsymbol{i}\boldsymbol{+}\boldsymbol{j}}\boldsymbol{)}\boldsymbol{,}$$

where $N=2m+1=7$ defines the window size, and the coefficients $c_{j}$derive from the local polynomial fit. This smoothing approach yields stable velocity and acceleration profiles that accurately retain the biomechanically relevant features of the suction event.

# 3 TABLES

**Table S1**. Morphometric data for hybrid Andrias specimens.

| **SPECIMEN** | **STL (cm)** | **SVL (cm)** | **HL (cm)** | **HW (cm)** | **M( kg)** | **Ontogenetic Stage** |
| --- | --- | --- | --- | --- | --- | --- |
| *1* | *76* | *49* | *11* | *10* | *1.7* | Adult |
| 2 | 47 | 31 | 7.2 | 7 | 1.35 | Subadult |
| 3 | 64 | 42 | 9.7 | 9.5 | 1.55 | Subadult |
| 4 | 69 | 45 | 10 | 10.5 | 1.7 | Subadult |
| *5* | *76* | *52* | *11.5* | *11.5* | *2.54* | Adult |
| *6* | *74.6* | *47.1* | *12.6* | *12.7* | *2.5* | Adult |
| *7* | *72.5* | *42* | *11.5* | *12* | *2.54* | Adult |
| *8* | *88* | *59* | *12.5* | *12.5* | *2.74* | Adult |
| 9 | 57 | 39 | 8.5 | 8.3 | 1.7 | Subadult |
| 10 | 65 | 44 | 10 | 10 | 1.6 | Subadult |
| 11 | 61 | 41 | 9.5 | 9 | 1.5 | Subadult |

**Table S2**. Experimental equipment and estimated uncertainties.

| **Equipment** | **Brand / Model** | **Measured variable** | **Units** | **Precision / Measurement error** |
| --- | --- | --- | --- | --- |
| High-speed camera | DITECT HAS-L1M | Frame rate (lateral), displacements, mandibular and hyoid angles | Hz, px, mm (after calibration) | ±1 Hz; tracking uncertainty treated separately (see Section 2.1) |
| Auxiliary frontal camera | Panasonic Lumix DMC-FZ300 | Frame rate (frontal), behavioural documentation | Hz, px | ±1 Hz; tracking uncertainty treated separately (see Section 2.1) |
| LED stroboscopic lighting system | High-stability LED panels | Light stability during high-speed recordings | Lux | Variation <5% per session |
| Digital balance | A&D EK-1200i | Prey mass | g | ±0.01 g |
| Digital caliper | Mitutoyo 500-196-30 | Prey total length | mm | ±0.1 mm |
| Calibration ruler / grid | Millimetric scale | Pixel–millimeter conversion | mm | ±0.05 mm |
| Water cooling system | GEX Cool Way BK-C220 | Water temperature | °C | ±0.5 °C |
| Filtration system | EHEIM 2213 | Water clarity, flow | — | Not applicable |
| Digital thermometer (verification) | High-precision digital thermometer | Water temperature verification | °C | ±0.1 °C |
| Digital hygrometer | Testo 608-H1 | Ambient relative humidity | %RH | ±2% RH |
| Acquisition software | Native DITECT / Lumix software | Recording control and acquisition | — | Not applicable |
| Analysis software | Tracker 6.3.3 / Python / SciPy / ImageJ | Displacements (x, y), angles, pixel-to-mm conversion | px, mm, degrees | ±0.1 mm (converted) |
| Velocity computation | Finite-difference approximation | Prey velocity magnitude | mm/s | Estimated from propagated positional uncertainty |
| Acceleration computation | Finite-difference approximation after signal smoothing | Signed tangential prey acceleration | mm/s² | Not quantified |
| Experimental tank | Glass aquarium (45 × 90 × 45 cm) | Filming environment, water depth, animal positioning | — | Not applicable |
| Camera support | Adjustable metal stand | Distance and height camera–tank | cm | ±1 mm |

# 4 TRIAL-LEVEL KINEMATIC RESULTS

**Figure S1.** Trial-level prey-motion profiles for all valid feeding events in each analysed specimen. Curves show normalized X displacement, Y displacement, prey velocity magnitude, and signed tangential prey acceleration. For specimens with multiple feeding trials, labels include the inferred display order in parentheses. The 1 g and 1 cm prey condition was assigned as T1 because this was the known first trial. Specimens with only one valid trial are shown without an order label. In the panel labels, “Speed” refers to prey velocity magnitude and “Acceleration” refers to signed tangential prey acceleration.

**Figure S2**. Magnitude of the two-dimensional prey acceleration vector during representative suction-feeding events in hybrid Andrias specimens. Prey acceleration magnitude was calculated as $a_{magnitude,prey}=\sqrt{a_{x}^{2}+a_{y}^{2}}$, where $a_{x}$and $a_{y}$represent the horizontal and vertical acceleration components, respectively. Unlike the signed tangential acceleration shown in Figure 7b, this quantity is necessarily non-negative and reflects changes in both the Prey velocity magnitude and direction of prey motion. Curves correspond to the same representative specimens shown in Figure 7.

# 5 REFERENCES

Challis, J.H., 1999. The appropriate filtering of biomechanical data. J. Appl. Biomech. 15, 308–321.

DITECT Corporation, 2022. HAS-L1 High-Speed Camera User Manual. DITECT Corporation, Tokyo, Japan.

Hatze, H., 1981. High-precision three-dimensional photogrammetric calibration and object space reconstruction techniques. J. Biomech. 14, 13–22.

Heiss, E., Natchev, N., Gumpenberger, M., Weissenbacher, A., Van Wassenbergh, S., 2013. Biomechanics and hydrodynamics of prey capture in the Chinese giant salamander reveal a high-performance jaw-powered suction feeding mechanism. J. R. Soc. Interface 10, 20121028. https://doi.org/10.1098/rsif.2012.1028

Lauder, G.V., 1982. Patterns of Evolution in the Feeding Mechanism of Actinopterygian Fishes. Am. Zool. 22, 275–285. https://doi.org/10.1093/icb/22.2.275

Savitzky, A., Golay, M.J.E., 1964. Smoothing and Differentiation of Data by Simplified Least Squares Procedures. Anal. Chem. 36, 1627–1639. https://doi.org/10.1021/ac60214a047

Winter, D.A., 2009. Biomechanics and Motor Control of Human Movement. John Wiley & Sons, Hoboken, NJ.

Zhang, Z., 2000. A flexible new technique for camera calibration. IEEE Trans. Pattern Anal. Mach. Intell. 22, 1330–1334.
